# Contrast-enhanced mammography versus breast MRI: a multidimensional cost-effectiveness analysis

**DOI:** 10.1007/s11547-025-02122-8

**Published:** 2025-10-21

**Authors:** Anna Colarieti, Alice Bonetti, Fiammetta Gianfrate, Silvia Attanasio, Anna Maria Rampi, Anna Clelia Lucia Gambaro, Alessandro Carriero

**Affiliations:** 1 Department of Translation Medicine, University of Eastern Piemonte UPO, Via Solaroli 17, 28100 Novara, Italy; 2 Radiology Department of Ospedale Maggiore della Carità, 28100 Novara, Italy

**Keywords:** Breast cancer, Contrast-enhanced mammography, Contrast-enhanced magnetic resonance imaging, Costs and cost analysis, Workflow

## Abstract

**Purpose:**

To assess the diagnostic concordance between contrast-enhanced mammography (CEM) and dynamic contrast-enhanced magnetic resonance imaging (DCE-MRI) in the preoperative staging of breast cancer (BCa) and to evaluate the clinical, operational, and economic implications of substituting CEM for MRI in concordant cases.

**Methods:**

This retrospective single-center study included 280 patients who underwent both CEM and DCE-MRI within 15 days. Two experienced breast radiologists independently evaluated imaging concordance based on lesion detection, characterization, and clinical interpretability. A cost minimization analysis was performed assuming equivalent diagnostic performance in concordant cases. Direct procedural costs were derived from regional reimbursement rates (€215.20 for DCE-MRI; €74.00 for CEM). Operational efficiency was assessed using institutional time-motion data, and average radiologist reporting times were included to model overall organizational impact.

**Results:**

Complete diagnostic concordance was observed in 190 cases (67.9%), while 78 (27.9%) showed that clinically relevant discordance and 12 (4.3%) were indeterminate. Substituting CEM for MRI in the concordant group yielded a projected total cost reduction of €26,828, representing a 44.9% decrease and €141.20 saved per patient. Procedural modeling indicated a net scanner time saving of 3,800 min (63 h and 20 min). CEM reporting required 15 min less per case on average, totaling 2,850 min (47 h and 30 min) in cumulative reading time saved.

**Conclusion:**

CEM demonstrated substantial diagnostic concordance with MRI in a majority of cases, with significant benefits in cost reduction and workflow efficiency. Targeted CEM integration may support more sustainable, value-based breast imaging without compromising diagnostic quality.

## Introduction

Breast dynamic contrast-enhanced magnetic resonance imaging (DCE-MRI) enables high-resolution three-dimensional and contrast-enhanced morpho-functional assessment of breast tissue and lesions, providing comprehensive characterization for diagnostic and investigational purposes [1, 2]. MRI plays a pivotal role across a broad range of clinical scenarios, including surveillance of high-risk individuals with BRCA1 or BRCA2 mutations [3], in patients with extremely dense breasts [4], staging of biopsy-proven malignancies, monitoring the treatment response to neoadjuvant chemotherapy, and problem-solving in cases of inconclusive conventional imaging [5]. It is particularly valuable for identifying non-mass enhancement, chest wall involvement, and multifocal or multicentric disease, all of which critically influence surgical planning and clinical management [6].

Despite these strengths, the widespread use of MRI is tempered by several practical limitations [7]. These include relatively high per-examination costs, lengthy examination durations (typically 25–45 min), constrained scanner availability, and contraindications in select patient groups—such as individuals with incompatible implants or claustrophobia [8]. These limitations necessitate exploration of alternative imaging strategies that maintain diagnostic efficacy while improving operational efficiency and patient accessibility.

Contrast-enhanced spectral mammography (CEM), approved by the US Food and Drug Administration in 2011 [9], represents an advanced breast imaging modality that has emerged as a clinically viable adjunct—and a potential alternative—to DCE-MRI. CEM utilizes dual-energy X-ray acquisition following intravenous administration of iodinated contrast agents to generate both low-energy (morphology-based) and recombined (functional) images [10, 11]. The latter selectively accentuate regions of neoangiogenesis by exploiting differential X-ray attenuation associated with increased vascular permeability and perfusion. By integrating anatomical and functional data, CEM approximates the diagnostic performance of DCE-MRI, while offering advantages including reduced acquisition time, lower cost, and broader availability [12]. A growing body of evidence supports the diagnostic non-inferiority of CEM relative to MRI for lesion detection, particularly in the local staging of biopsy-proven breast malignancies [13, 14].

However, while diagnostic performance metrics are increasingly available, there remains a paucity of data evaluating the broader healthcare impact of integrating CEM into routine practice—particularly with regard to cost-efficiency, time savings, and operational throughput. These organizational metrics are critical in modern radiology departments, where imaging demand often exceeds available resources.

This study aims to systematically assess the diagnostic concordance between CEM and DCE-MRI in a large cohort of patients undergoing both modalities for preoperative staging of breast cancer (BCa) and to conduct a comprehensive comparative analysis of procedural time metrics and direct cost parameters, with the objective of quantifying the potential clinical, operational, and economic implications of implementing CEM as a substitute for DCE-MRI in diagnostically concordant cases.

## Materials and methods

### Study design and patient population

This single-center, retrospective observational study was conducted at a high-volume tertiary care academic radiology department and was approved by the institutional review board. All participants provided written informed consent for the use of their anonymized clinical and imaging data for research purposes (Num. 118/20). The study was conducted in accordance with the ethical standards of the institutional and national research committees and with the principles outlined in the Declaration of Helsinki. Between February 2018 and May 2025, all consecutive female patients referred for preoperative staging of histologically confirmed BCa who underwent both CEM and DCE-MRI were screened for eligibility.

A total of 280 patients met the inclusion criteria, which comprised: (1) availability of both CEM and DCE-MRI examinations acquired for the same clinical indication (preoperative staging), (2) both imaging modalities performed within a maximum interval of 15 days to minimize interval disease progression or treatment effects, and (3) technical adequacy and diagnostic quality of both examinations, as independently verified by two board-certified breast radiologists with a minimum of 10 years of experience.

Patients were excluded if they had: (1) incomplete imaging datasets, suboptimal image quality precluding confident interpretation, (2) prior neoadjuvant therapy between the two examinations, or (3) known contraindications to contrast agents. All imaging was performed according to standardized institutional protocols, with intravenous administration of iodinated contrast for CEM and gadolinium-based contrast for DCE-MRI.

### Imaging protocols

#### CEM

CEM examinations were performed using a full-field digital mammography system (Selenia Dimensions®, Hologic Inc., Marlborough, MA, USA). Prior to image acquisition, a peripheral venous catheter was inserted in the forearm. A single intravenous bolus of a low-osmolar non-ionic iodinated contrast agent (Iomeron® 350 mgI/mL, Bracco Imaging, Milan, Italy) was administered via a dual-head power injector (EmpowerCTA®, Bracco Injeneering, Italy) at a flow rate of 2–3 mL/s, followed by a 20 mL saline flush. The contrast dose was weight based, at 1.5 mL/kg, with a maximum volume not exceeding 110 mL. The injection was performed with the patient seated.

Image acquisition commenced one minute following contrast administration, beginning with the clinically suspected breast. Two dual-energy mammographic projections—craniocaudal (CC) and mediolateral oblique (MLO)—were acquired in that order using the tomosynthesis modality. Subsequently, delayed 2D projections (CC and MLO) were acquired in the same order, starting at the seventh minute post-injection.

#### DCE-MRI

MRI examinations were performed using a 3.0-Tesla superconducting magnet system (Ingenia, Philips Healthcare, Best, The Netherlands) equipped with a dedicated multi-channel phased-array breast coil. All patients were imaged in the prone position. The imaging protocol included axial and sagittal T2-weighted fast spin-echo sequences, a pre-contrast axial T1-weighted 3D gradient-echo sequence, and a dynamic post-contrast series composed of six sequential acquisitions. Diffusion-weighted imaging (DWI) was also performed using b-values of 0 and 850 s/mm^2^, with corresponding apparent diffusion coefficient (ADC) maps automatically generated.

A gadolinium-based macrocyclic contrast agent (gadobutrol, Gadovist®, Bayer Healthcare, Berlin, Germany) was administered intravenously at a dose of 1 mL/kg body weight using a dedicated contrast injector (Spectris Solaris EP®, Bayer Healthcare) at an infusion rate of 2 mL/min, followed by a saline flush. Total examination time was approximately 25 min. The technical parameters of the sequences are summarized in Table 1.

**Table 1:** Summary of key DCE-MRI acquisition parameters

Sequence	TR/TE (ms)	Flip angle (°)	Slice thickness (mm)	FOV (mm)	Matrix
T2-weighted FSE	4000–6000/80–120	90	3–4	300–360 (adjusted to breast size)	~ 320 × 256
Diffusion-Weighted Imaging (DWI)	5000–8000/60–100	90	3–4	300–360	~ 128 × 128
T1-weighted (pre-contrast)	600–800/10–15	70–90	3	300–360	~ 256 × 192
Dynamic Contrast-Enhanced (DCE) T1W GRE	4–6/1.5–2.5	10–15	1–2	300–360	~ 384 × 384

The time between CEM and MRI was limited to a maximum of 15 days to ensure temporal proximity and clinical comparability. All images were reviewed by two experienced breast radiologists (20 and 12 years of experience in breast imaging, respectively) blinded to clinical outcomes and other imaging findings.

### Image interpretation and modality concordance assessment

All imaging studies were independently reviewed by two experienced breast radiologists, each with over 10 years of subspecialty experience in breast imaging. Readers were blinded to all clinical data, histopathologic findings, and to each other’s interpretations. The evaluation process was standardized and performed in a randomized order to minimize interpretive bias.

Lesion assessment was based on established criteria, including lesion detection (presence/absence), localization, morphological features (mass vs. non-mass enhancement, shape, margins, internal enhancement patterns), and maximum lesion diameter. BI-RADS descriptors were applied where applicable, and measurements were obtained in the axial plane.

Concordance between CEM and DCE-MRI was assessed on a per-patient basis and categorized into three predefined classes:

- Complete concordance: defined as agreement between CEM and DCE-MRI in terms of lesion presence, number of lesion(s), location, morphology, and size (± 5 mm difference), with imaging findings expected to result in concordant clinical decision-making (e.g., surgical planning, biopsy targeting). This category reflects interchangeability in terms of diagnostic and therapeutic implications.
- Non-evaluable/indeterminate: applied when findings were equivocal or insufficiently characterized to permit a confident comparative interpretation.
- Clinically relevant discordance: defined as discrepancies in lesion visibility, number, anatomical localization, morphological characterization, or extent of disease, such that clinical management would likely differ between the two modalities.

Concordance classification was determined by consensus after independent review. In cases of initial disagreement, a consensus meeting was held between the two radiologists to resolve classification, without recourse to clinical or histopathological information.

**Fig. 1:**
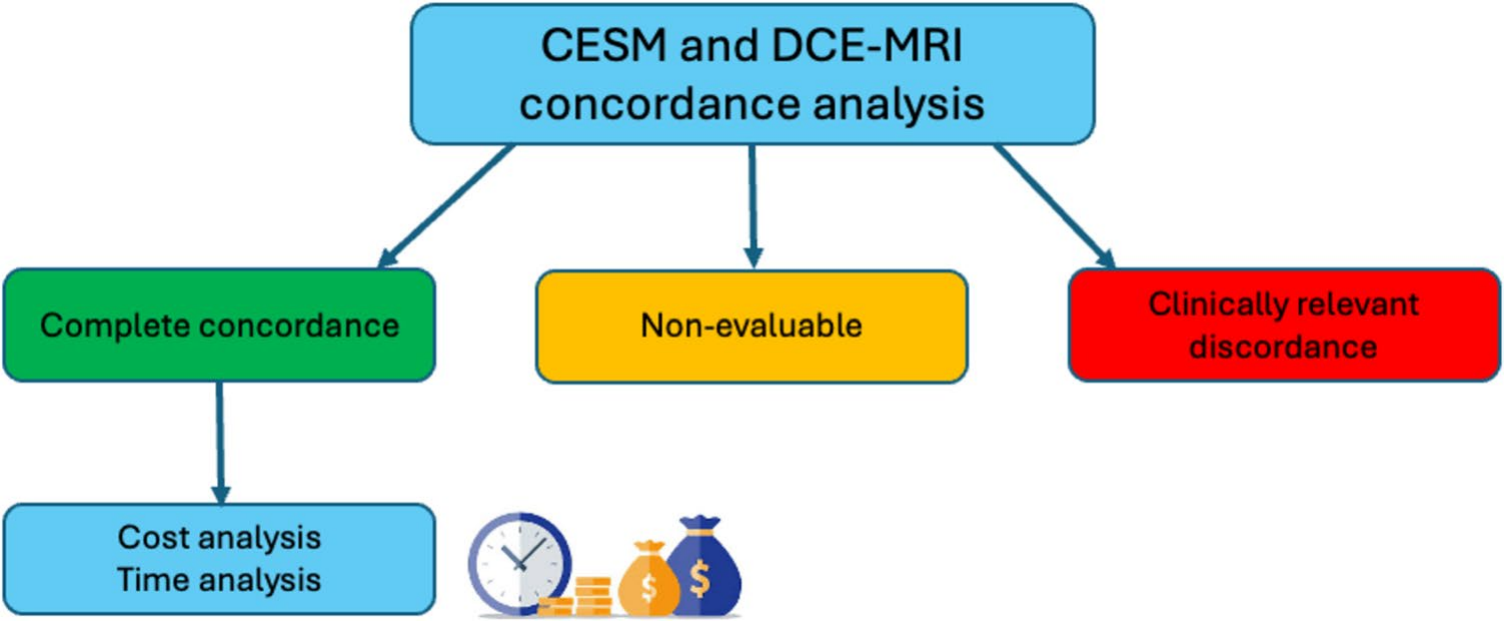
Schematic representation of the study workflow. Patients undergoing both CEM and DCE-MRI were evaluated on a per-patient basis and classified into one of three predefined concordance categories: complete concordance, clinically relevant discordance, or non-evaluable/indeterminate. Only cases with complete concordance were included in subsequent health economic and workflow analyses

Only cases classified as complete concordance were included in the downstream health economic and workflow analysis, under the assumption that substituting MRI with CEM in these cases would not compromise diagnostic accuracy or alter patient management pathways. The study workflow is summarized in Fig. 1.

To evaluate the reproducibility of imaging interpretation, inter-reader agreement between the two breast radiologists was quantified using **Cohen’s kappa (κ) statistics.** Agreement was calculated separately for three diagnostic domains: (1) lesion detection (presence/absence), (2) morphological characterization (mass vs. non-mass enhancement, margins, internal enhancement patterns), and (3) assessment of disease extent (maximum diameter, multifocality, multicentricity).

Kappa coefficients were interpreted according to the widely adopted Landis and Koch benchmarks: ≤ 0.20, poor; 0.21–0.40, fair; 0.41–0.60, moderate; 0.61–0.80, substantial; and ≥ 0.81, almost perfect agreement. Ninety-five percent confidence intervals (95% CI) were computed for each *κ* estimate to account for sampling variability.

### Economic and organizational impact analysis

A cost minimization analysis was carried out under the assumption of equivalent diagnostic performance between CEM and DCE-MRI in patients with complete imaging concordance. This approach was based on the principle that, when clinical outcomes remain unaffected, the most economically efficient strategy should be prioritized. Direct procedural costs were estimated using regionally applicable outpatient reimbursement rates, with the Piedmont rate applied for DCE-MRI (€215.20 per examination) and the Lombardy rate used as a proxy for CEM (€74 per examination), as CEM is not yet reimbursed in Piedmont. The analysis focused on patients classified as concordant and estimated total cost savings by multiplying the number of eligible cases by the unit cost difference between the two modalities.

### Operational efficiency modeling

To quantify potential workflow improvements, an organizational time-efficiency model was applied. Modality-specific average procedural durations were derived from institutional time-motion data, reflecting typical room occupancy per-patient encounter. The average procedural time for DCE-MRI was estimated at 30 min, while CEM required approximately 10 min per case. Total procedural time savings were calculated across the concordant cohort, representing cumulative reductions in scanner occupancy. All economic and temporal outcomes are expressed in both absolute and relative terms, with the aim of informing cost-containment strategies and operational decision-making in breast imaging services.

### Reporting time analysis

In addition to procedural acquisition times, radiologist interpretation and reporting times were incorporated into the organizational efficiency assessment. These estimates were based on institutional averages obtained from experienced breast radiologists routinely interpreting both CEM and DCE-MRI examinations. Reporting time for DCE-MRI was estimated to range between 25 and 40 min per examination, while CEM reporting was typically completed within 15–20 min.

## Results

### Image interpretation and modality concordance assessment

Diagnostic concordance between CEM and DCE-MRI was assessed in the final study cohort comprising 280 patients who underwent both imaging modalities within a 15-day interval as part of their preoperative staging for breast cancer. Demographic and clinical characteristics of the study cohort are summarized in Table 2.

**Table 2:** Patients’ characteristics

Number of patients	280
Age (mean years ± SD)	Mean 58.0 ± 11.8 (range 32–85)
Histology	Ductal carcinoma: 112 (40.0%) Invasive carcinoma: 91 (32.5%) Lobular carcinoma: 57 (20.4%) In situ carcinoma: 6 (2.1.%) Other: 14 (5.0%)
Focality	Monofocal: 116 (59.5%) Bifocal: 20 (10.2%) Multifocal: 24 (12.3%) Multicentric/contralateral: 6 (3.1%) Other/unspecified: 14 (7.0%)

**Fig. 2:**
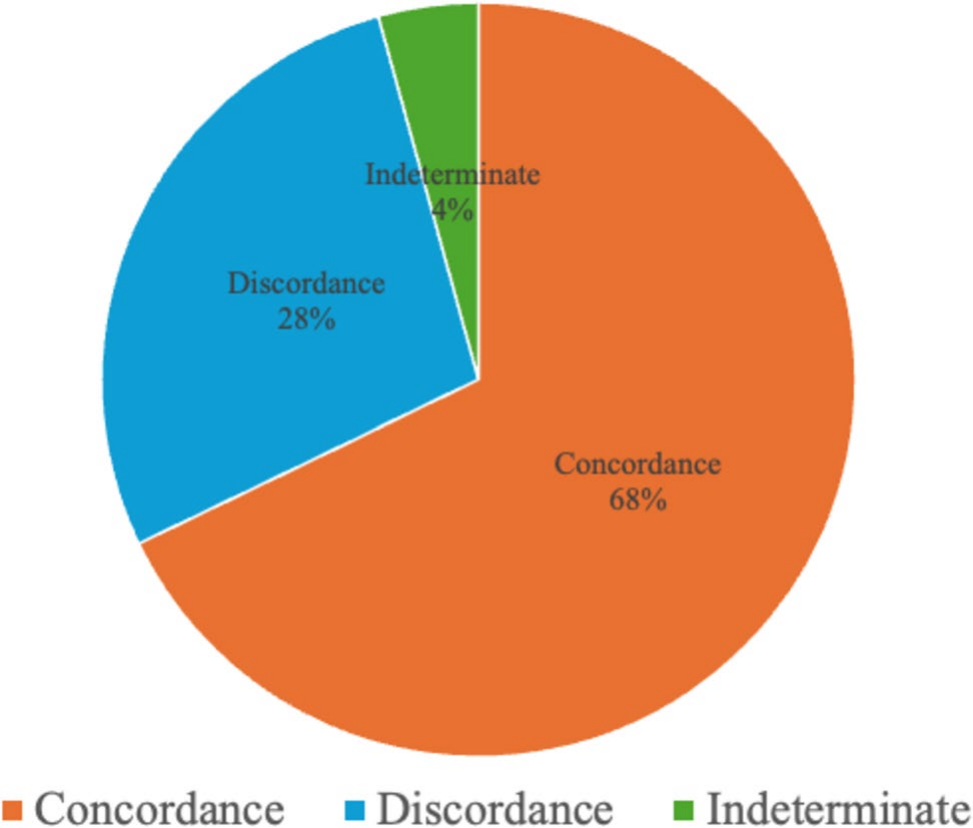
Distribution of concordance classifications between CEM and DCE-MRI on a per-patient basis. Among the evaluated cases, 68% demonstrated complete concordance (orange), 28% showed clinically relevant discordance (light blue), and 4% were deemed non-evaluable or indeterminate (green)

Image interpretation was independently performed by two breast radiologists, with consensus for discordant cases. Complete concordance was observed in 190 patients (67.9%), clinically meaningful discordance in 78 (27.9%), and indeterminate findings in 12 (4.3%) (Fig. 2).

Image interpretation was independently performed by two breast radiologists, and discordant cases were subsequently reviewed to reach a consensus classification. Among the cohort, complete concordance between CEM and DCE-MRI—defined as agreement in lesion detection, morphology, location, and extent, with no expected impact on clinical management—was observed in 190 patients, corresponding to 67.9% of cases. Clinically meaningful discordance, which entailed discrepancies likely to influence diagnostic or therapeutic decisions, was documented in 78 patients (27.9%). The remaining 12 cases (4.3%) were categorized as indeterminate due to inconclusive or equivocal findings on one or both modalities (Fig. 2).

### Inter-reader agreement

The inter-reader reproducibility of imaging interpretation was high across all diagnostic domains.

- Lesion detection demonstrated almost perfect agreement, with *κ* = 0.82 (95% CI 0.77–0.87).
- Morphological characterization showed substantial agreement, with *κ* = 0.78 (95% CI 0.72–0.84).
- Extent of disease assessment achieved substantial agreement, with *κ* = 0.74 (95% CI 0.68–0.80).

### Economic and organizational impact analysis

This analysis was performed on the concordant subgroup, under the assumption of equivalent diagnostic utility.

**Fig. 3:**
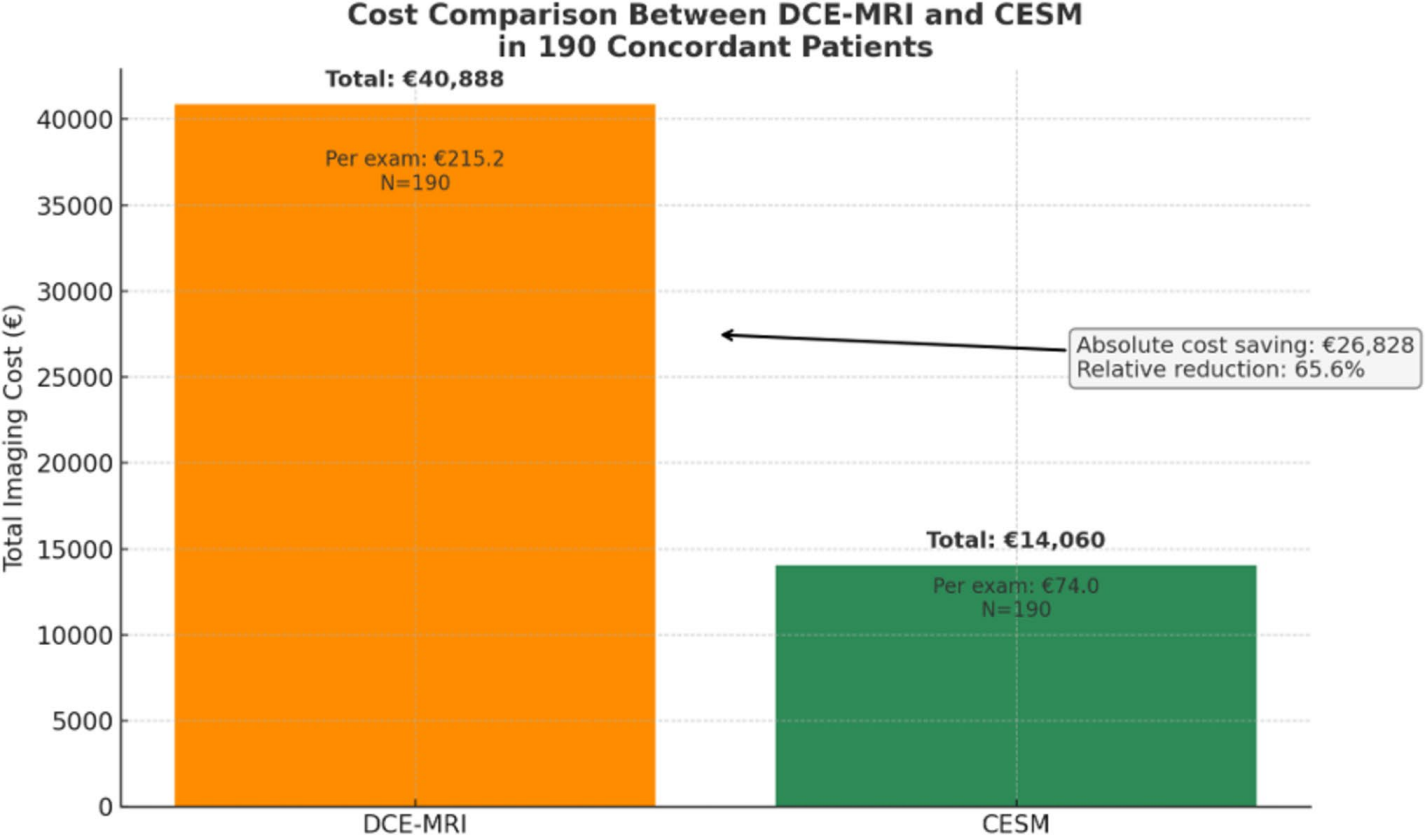
Detailed cost comparison between DCE-MRI and CEM. Bars represent total imaging expenditures based on standardized per-exam reimbursement rates

Direct procedural costs were €215.20 per DCE-MRI and €74.00 per CEM. Substitution of DCE-MRI with CEM yielded a projected saving of €26,828, corresponding to €141.20 per patient and a 44.9% reduction in imaging-related expenditure (Fig. 3).

### Operational efficiency modeling

**Fig. 4:**
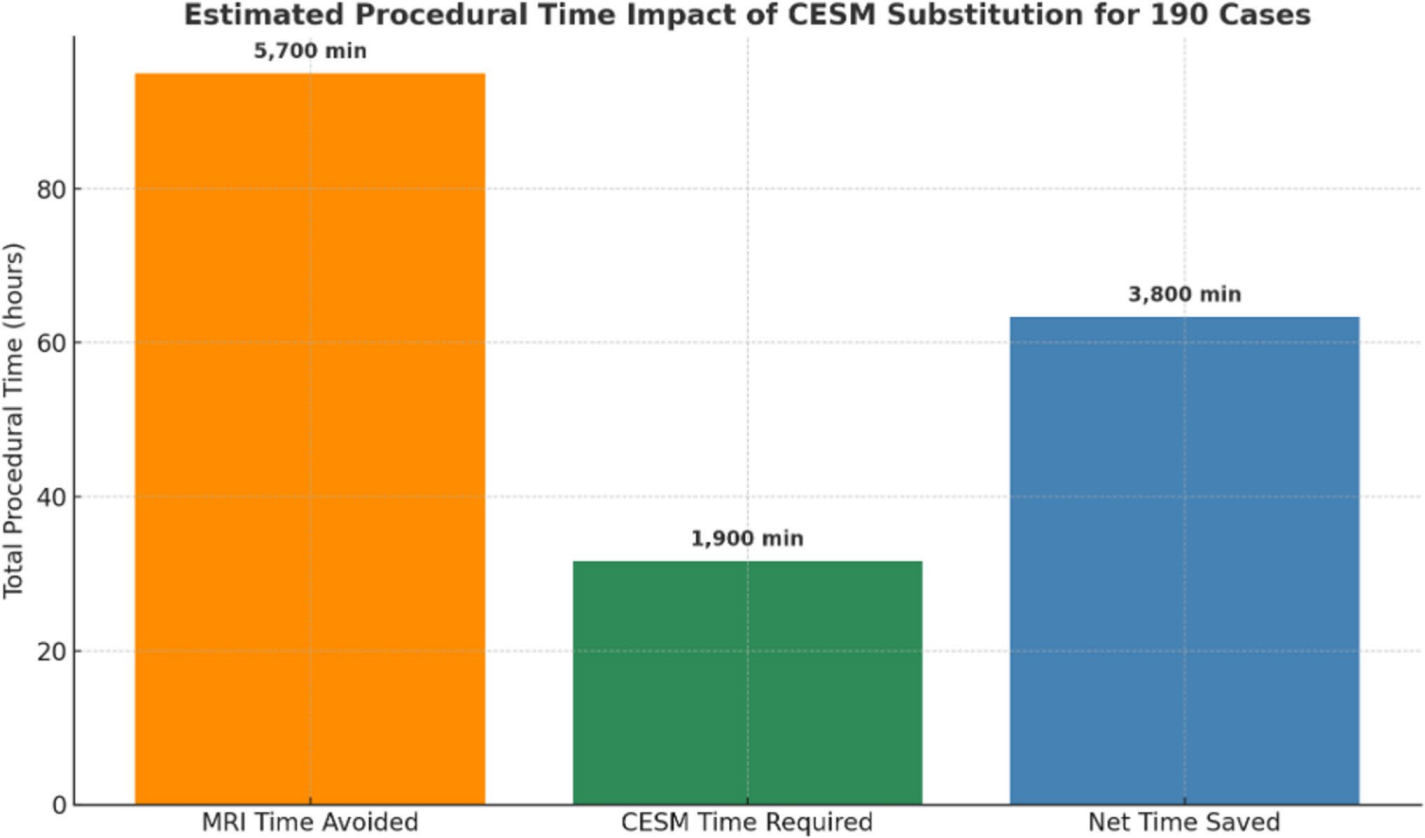
Estimated impact of substituting DCE-MRI and CEM. Data are based on average institutional exam durations of 30 min for DCE-MRI and 10 min for CEM

Average procedure duration was 30 min for DCE-MRI and 10 min for CEM. Replacing concordant DCE-MRI examinations with CEM resulted in a net scanner time saving of 3800 min (63 h and 20 min) (Fig. 4).

### Reporting time analysis

In the cohort of 190 diagnostically concordant cases, CEM consistently required shorter interpretation times compared with DCE-MRI. The mean reporting time was **32 min and 30 s per case** for DCE-MRI and **17 min and 30 s per case** for CEM, resulting in an average per-case saving of **15 min**. When extrapolated to the entire concordant cohort, this corresponded to a total reduction of 2850 min, equivalent to 47 h and 30 min.

**Fig. 5:**
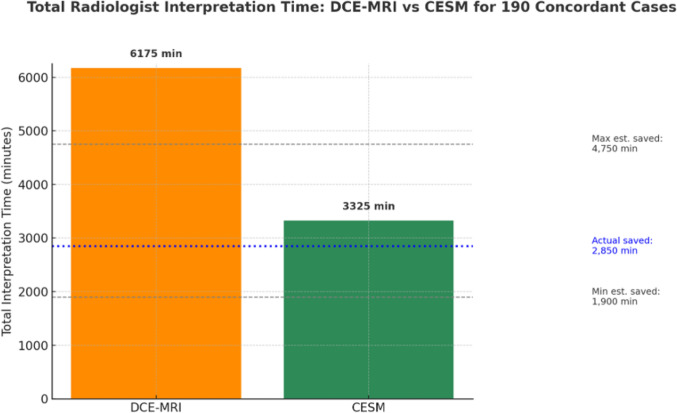
Comparative analysis of radiologist interpretation time for DCE-MRI and CEM

Sensitivity analysis, based on the institutional ranges of interpretation times (**25 to 40 min for DCE-MRI** and **15 to 20 min for CEM**), confirmed the robustness of these findings. Under conservative assumptions, total reporting time savings across the concordant cohort ranged from **1900 min (31 h and 40 min)** to **4750 min (79 h and 10 min)** (Fig. 5).

## Discussion

This study provides evidence supporting the clinical reliability of CEM as a diagnostic alternative and operationally advantageous to DCE-MRI in the preoperative evaluation of BCa. In a cohort of 280 patients undergoing both modalities, there was a complete diagnostic concordance rate of 67.9%, defined by agreement in lesion detection, morphological characterization, and extent of disease. These findings add to the growing body of literature validating CEM as a high-performing modality in breast imaging, particularly in patients with unifocal disease or contraindications to MRI. While DCE-MRI retains an essential role in complex diagnostic scenarios—such as in cases of invasive lobular carcinoma, suspected multifocal or multicentric disease, and high-risk screening—our data suggest that CEM yields comparable actionable information for a substantial proportion of patients, in concordance with the actual literature [15, 16]. The diagnostic implications are notable, particularly given the increasing need for scalable, high-throughput imaging pathways in oncology care.

To the best of our knowledge, this is the first study to move beyond diagnostic performance and provide a comprehensive evaluation of the economic and operational value of integrating CEM into imaging workflows applying European reimbursement structures and integrating an organizational efficiency assessment, thereby expanding the evidence base on the potential economic role of CEM. In the diagnostically concordant subgroup, substitution of MRI with CEM resulted in a 44.9% per-patient reduction in imaging-related costs, translating to €26,828 in cumulative direct savings. Cost minimization is crucial in publicly funded healthcare systems, where imaging services face financial limits and prioritization. Extrapolation of this model to broader patient populations or regional imaging networks could yield significant cumulative savings while preserving clinical efficacy. The substitution of MRI with CEM in concordant cases thus represents a significant opportunity for resource optimization in breast imaging, with preserved diagnostic accuracy. While our study demonstrates that CEM and MRI show concordant findings in a relevant proportion of patients, it was not designed to systematically identify predictors of concordance. Preliminary observations suggest that concordance is more frequent in unifocal invasive ductal carcinomas, whereas discordance is associated with multifocal/multicentric disease, lobular histology, and dense breast parenchyma, as well as variability in background parenchymal enhancement [17], 18. Prospective studies are required to formally define patient- and lesion-related factors that could guide the safe substitution of MRI with CEM in selected clinical settings.

From a healthcare resource optimization perspective, the operational impact of substituting DCE-MRI with CEM is both quantifiable and clinically significant. The replacement of 190 MRI examinations with CEM resulted in an overall reduction of approximately 5700 min (95 h) of MRI scanner usage. After adjusting for the total procedural time required to perform the corresponding CEM studies—estimated at 1900 min—the net time savings per patient amount to 3800 min (63 h and 20 min). This liberated scanner capacity represents a critical opportunity for the reallocation of imaging resources to address increasing clinical demand and mitigate existing procedural backlogs, particularly in high-complexity imaging pathways. Based on institutional mean scan durations for commonly performed MRI examinations, this net gain in scanner availability could theoretically enable the execution of a substantial number of additional studies, including approximately 190 brain MRI examinations (mean duration per examination ~ 30 min), 228 multiparametric prostate MRI studies (mean duration per examination ~ 25 min), 190 neck MRI studies (mean duration per examination ~ 30 min), 142 upper abdominal MRI examinations with contrast medium (mean duration per examination ~ 40 min), and approximately 114 cardiac MRI examinations (mean duration per examination ~ 50 min). These projections underscore the potential of CEM to act not only as a clinically viable alternative in appropriate diagnostic settings but also as a strategic lever to enhance the throughput and responsiveness of MRI services in tertiary care environments. These redeployments are not merely logistical conveniences—they represent meaningful clinical capacity expansion, particularly in oncologic care pathways where imaging delays can critically impact diagnostic timelines, treatment initiation, and overall patient outcomes.

Moreover, CEM’s shorter acquisition time, lower technical demands, and broader availability enhance scheduling flexibility and throughput. When combined with its strong diagnostic yield, these advantages position CEM as a high-value modality in stratified imaging strategies. In this context, our findings support the implementation of a triage-based, value-driven imaging model in which CEM serves as a frontline or problem-solving tool for selected patients, preserving MRI for cases where it adds incremental diagnostic benefit.

An additional advantage of CEM lies in its greater territorial availability compared to breast MRI. While DCE-MRI requires dedicated infrastructure, specialized personnel, and stringent scheduling logistics often limited to tertiary centers, CEM systems are more broadly distributed across community and peripheral hospitals. This wider accessibility enables more equitable diagnostic service delivery, particularly in underserved or rural regions where MRI access may be constrained.

In addition, CEM required 15 fewer minutes per case for reporting, translating to a total reduction of 2850 min (47 h and 30 min) in interpretive time across the concordant cohort. Incorporating this metric into the operational model further underscores the systemic benefit of CEM implementation—not only in terms of scanner availability but also in optimizing radiologist workload and reporting efficiency. Furthermore, CEM demonstrates higher patient acceptance due to its shorter acquisition time, lower levels of procedural anxiety, and absence of confined environments typically associated with MRI-related discomfort. This is especially relevant for patients with claustrophobia, implanted medical devices, or limited tolerance for prolonged examinations [19]. From a professional standpoint, the interpretive complexity of CEM is generally lower than that of breast MRI. Radiologists can more readily acquire and apply CEM interpretive skills due to the modality’s reliance on conventional mammographic projection geometry and contrast kinetics, which are more intuitive than the multiparametric and high-dimensional data typical of MRI. This facilitates faster learning curves and more consistent diagnostic performance, particularly in settings where sub-specialization in breast imaging is limited [20]. Collectively, these factors further strengthen the case for CEM as a pragmatic and scalable solution for enhancing the reach and efficiency of BCa diagnostic pathways.

This modeling aimed to estimate potential improvements in imaging suite throughput, resource reallocation capacity, and system-level scheduling efficiency. Taken together, these results provide a multidimensional assessment—diagnostic, economic, and organizational—of a CEM-first strategy in the presurgical staging of Bca. While previous studies have demonstrated the diagnostic comparability of CEM and MRI, this analysis uniquely quantifies the downstream impacts on healthcare delivery infrastructure, making a compelling case for the selective integration of CEM as part of a value-based breast imaging paradigm.

Despite the strengths of this analysis, some limitations must be acknowledged. First, its retrospective design and single-institution setting may introduce inherent selection and referral biases. Second, the study relied on imaging concordance as a surrogate marker of diagnostic equivalence; no direct assessment of clinical outcomes (e.g., surgical margin status, recurrence, or survival) was performed. While concordance provides a pragmatic metric for modeling resource allocation, it does not fully substitute for histopathological correlation or patient-centered endpoints, and the substitution of MRI with CEM remains a hypothetical model that requires validation through prospective studies including clinical outcomes and long-term follow-up. Finally, imaging acquisition protocols and interpretive workflows may vary across institutions, which could affect the reproducibility of results in different clinical environments. Prospective, multi-center studies with standardized protocols and integrated clinical outcome measures are needed to validate these findings and support broader implementation of CEM-based substitution strategies.

In conclusion, CEM demonstrated a high diagnostic concordance with DCE-MRI in the preoperative staging of BCa. Beyond its diagnostic performance, CEM offers significant advantages in cost-effectiveness, operational efficiency, interpretive simplicity, and patient acceptability. Its broader availability and more accessible implementation make CEM a pragmatic and scalable alternative in selected patient populations. These findings support the selective incorporation of CEM into value-based breast imaging strategies, with the potential to enhance the quality, equity, and sustainability of oncologic imaging services, contributing to more efficient use of diagnostic resources within value-based healthcare frameworks.

## Data Availability

The datasets generated and analyzed during the present study are not publicly available due to institutional policies and patient privacy regulations. However, anonymized data may be made available by the corresponding author upon reasonable request for academic and non-commercial research purposes, subject to appropriate data use agreements.

## Abbreviations

BCa: Breast cancer
CEM: Contrast-enhanced spectral mammography
DCE-MRI: Dynamic contrast-enhanced magnetic resonance imaging

## Declarations

### Human and animals rights

This research does not involve human participants and/or animals.
